# Minimum sample sizes for invasion genomics: Empirical investigation in an invasive whitefly

**DOI:** 10.1002/ece3.5677

**Published:** 2019-10-02

**Authors:** Wan‐Mei Qu, Ni Liang, Zi‐Ku Wu, You‐Gang Zhao, Dong Chu

**Affiliations:** ^1^ Key Lab of Integrated Crop Pest Management of Shandong Province College of Plant Health and Medicine Qingdao Agricultural University Qingdao China; ^2^ Science and Information College Qingdao Agricultural University Qingdao China

**Keywords:** 2b‐RAD, empirical study, invasive species, population genomics

## Abstract

Analysis of population genetics provides insights into the evolutionary processes, among which the sample size choice is per se a crucial issue in the analysis. Genome‐wide high‐throughput techniques based on RADseq have been increasingly used in studies on the population genomics of invasive species. However, there is little information available regarding optimal sample sizes for analyzing population genomics of invasive species. In this study, we first use type IIB endonucleases restriction site‐associated DNA (2b‐RAD) to mine thousands of single nucleotide polymorphisms (SNPs) for native and introduced populations in Q1 clade (SPB and 17JN) and Q2 clade (ISQ and UAS0601) of the whitefly, *Bemisia tabaci* (Gennadius) MED (also known as *B. tabaci* biotype Q). Then, we used resampling techniques to create simulated populations with a random subset of individuals and 3,000 SNPs to determine how many individuals should be sampled for accurate estimates of intra‐ and interpopulation genetic diversity. We calculated the intrapopulation genetic diversity parameters (unbiased expected heterozygosity, observed heterozygosity, and the number of effect alleles) and pairwise genetic differentiation *F*_ST_; finally, an ad hoc statistic, Δ*K*, was used to determine the optimal value. Our results showed that a sample size greater than four individuals (*n* ≥ 4) has little impact on estimates of genetic diversity within whitefly populations; moreover, precise estimate of *F*_ST_ can be easily achieved at a very small simple size (*n* = 3 or 4). Our results will provide in‐depth understanding of the optimization of sampling schemes in population genomics of invasive species.

## Graphical abstract

We evaluated the optimal sample sizes for population genomics of invasive species. Our results showed that a sample size greater than four individuals (*n* ≥ 4) has little impact on estimates of genetic diversity within whitefly populations; moreover, precise estimate of *F*_ST_ can be easily achieved at a very small simple size (*n* = 3 or 4).

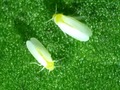

## 1 INTRODUCTION

Analysis of population genetics provides insights into the evolutionary processes, among which the sample size selection is per se a crucial issue in the analysis (Hale, Burg, & Steeves, 2012; Nazareno, Bemmels, Dick, & Lohmann, 2017; Willing, Dreyer, & Oosterhout, 2012). Limited sampling can lead to ambiguous or negative results (Hale et al., 2012). Generally, larger sample sizes are thought to be necessary when conducting an analysis of population genetics. Obtaining larger samples, however, can result in extra expense and wasted analytical time (Willing et al., 2012).

Recent studies have shown that analyzing population genetics can be conducted with small sample sizes when using a large number of single nucleotide polymorphisms (SNP; Jeffries et al., 2016; Nazareno et al., 2017). For instance, a recent empirical study by Jeffries et al. (2016) demonstrated that genome‐wide high‐throughput techniques based on restriction site‐associated DNA sequencing (RADseq), which can provide large numbers of SNPs, could obtain a finer population structure and stronger patterns of isolation‐by‐distance (IBD) than microsatellites with only 17.6% of samples. The techniques based on RADseq include original RAD, double‐digest RAD (ddRAD), type IIB endonucleases restriction site‐associated DNA (2b‐RAD), and ezRAD (Andrews, Good, Miller, Luikart, & Hohenlohe, 2016; Davey et al., 2011). In recent years, the RADseq technique has been widely used in the study of population genomics (Blanco‐Bercial & Bucklin, 2016; Wosula, Chen, Fei, & Legg, 2017), with the minimum sample size for population genomics being evaluated based on simulation (Willing et al., 2012) or empirical analysis (Nazareno et al., 2017). For example, by estimating the genetic differentiation (*F*_ST_) a simulation analysis has shown that sample size can be reduced to four to six individuals when using a large number of SNPs (Willing et al., 2012). An empirical study also has found that the genetic diversity and genetic differentiation (*F*_ST_) in a plant species can be accurately estimated from 6 to 8 individual plants using SNPs based on ddRADseq (Nazareno et al., 2017). Inconsistencies that have been noted between the results obtained by simulation and by empirical analysis suggest that it may be necessary that the minimum sample size be assessed for each species. For example, most insects have a high dispersal ability and thus high gene flow between different populations. This is especially true in migratory insects (Li et al., 2016; Yin et al., 2017) and is obviously different from the empirical analysis used on plant populations. In addition, many other factors including demographic history, intrinsic life‐history traits, and overall population characteristics should also be considered (Nazareno et al., 2017). Thus, more empirical studies should be performed to determine ideal sampling schemes.

Analyzing the population genetics of invasive species can determine the degree of population connectivity and the source of invasion, as well as assess the potential for spread of non‐native species (Geller, Darling, & Carlton, 2010). Invasion genetics can be shaped by several factors including the number of individuals introduced, the diversity and differentiation of the source population(s), multiple introductions, genetic drift, and natural dispersal (Lallias et al., 2015). Based on the data of microsatellite loci, a recent study by Lombaert, Guillemaud, and Deleury (2018) addressed the use of STRUCTURE software and showed that the analysis of invasion genetics may be misleading when the native population has a low level of diversity or when a large number of loci are used, which implies that the invasion of species may affect the analysis of population genetics. In recent years, genome‐wide high‐throughput techniques based on RADseq have been increasingly used to study this topic (Elfekih et al., 2018; Resh, Galaska, & Mahon, 2018; Yi et al., 2018). However, little is currently known regarding the optimal sample sizes needed for analyzing the population genomics of invasive species.

To determine the optimal sample sizes for population genomics of invasive species, we first used resampling techniques to create simulated populations with a random subset of individuals from the populations of invasive species, *Bemisia tabaci* (Gennadius) MED (also known as *B. tabaci* biotype Q—hereafter referred to as *B. tabaci* Q); *B. tabaci* Q, a member of the *B. tabaci* species complex, is a highly invasive species that has spread from its origin in the countries bordering the Mediterranean Basin to at least ten additional countries in the past two decades, including China and the United States (De Barro, Liu, Boykin, & Dinsdale, 2011; Gnankiné et al., 2013). We then calculated the intrapopulation genetic diversity parameters (unbiased expected heterozygosity, observed heterozygosity, and expected heterozygosity) and the pairwise genetic differentiation *F*_ST_, respectively; finally, an ad hoc statistic, Δ*K*, was used to judge the minimum sample sizes for intra‐ and interpopulation genetic diversity (Evanno, Regnaut, & Goudet, 2005). The Δ*K* means the second‐order rate of change in the likelihood function with respect to *K*; after the Δ*K* reaches the peak value, increasing the *K* value appeals to have little impact on the genetic diversity parameters.

## 2 MATERIALS AND METHODS

### 2.1 Whitefly samples and DNA extraction

The samples of *B. tabaci* used in this study were obtained from four locations: Israel (code: ISQ), the United States (code: USA0601), Spain (code: SPB), and Jinan of China (code: 17JN). While *B. tabaci* Q is a native species in the Mediterranean regions (Israel and Spain), it is an introduced species in the United States and China. Ten females were selected for sequencing from each population. The specimens were then preserved in 95% ethanol and stored at −80°C until DNA extraction. The DNA of individuals was extracted using a TIAMamp Micro DNA Kit (Tiangen Biotech [Beijing] Co., Ltd.) according to the protocol. Prior to sequencing, the DNA concentration of each individual was quantified by NanoDrop and Agarose Gel.

### 2.2 Species identification

The mitochondrial cytochrome c oxidase I (mtCOI) gene was amplified using forward primer 2195MF (5′‐CTGGTTYTTTGGTCATCCRGARGT‐3′; Simon et al., 1994) and newly designed reverse primer 2830R (5′‐CAATCAGCATAATCTGAATATCG‐3′) which amplified a 635 bp fragment. The PCRs were performed in 20 µl solutions containing 1 × buffer, 0.32 mM of each dNTP, 1.0 mM of each primer, 1.0 unit of Taq DNA polymerase, and 2 µl of template DNA. PCRs were performed under the following conditions: initial denaturation at 95°C for 5 min, followed by 35 cycles of 1 min at 94°C for denaturation, 1 min at 54°C, for annealing and 1 min at 72°C for elongation, and final extension at 72°C for 5 min. The PCR products were electrophoresed in a 1.0% agarose gel in TAE and were sequenced bidirectionally. The similarities of sequences were detected using the BLAST algorithm of NCBI.

Based on the fragments as defined by De Barro and Ahmed (2011), using *Trialeurofes vaporariorum* mtCOI gene (GenBank ID: AF418672) as an outgroup, multiple mtCOI sequences were aligned using the ClustalW algorithm in MEGA7 (Kumar, Stecher, & Tamura, 2016; Thompson, Higgins, & Gibson, 1994); then, the sequences were trimmed to 482 bp. MtCOI‐based distances were calculated with the Kimura 2‐parameter model of MEGA7. The phylogenetic trees were built using the maximum likelihood (ML) method with bootstraps of 1,000 replications in MEGA7 (Kumar et al., 2016).

### 2.3 2b‐RAD Library preparation and sequencing

The 2b‐RAD libraries were prepared at Qingdao OE Biotech Co., Ltd., as described in Wang, Meyer, Mckay, and Matz (2012). For each sample, 100–200 ng of genomic DNA was digested by 1 U *Bsa*XI (New England Biolabs, cat. no. R0609) in a 15 µl reaction at 37°C for 45 min. A 4 µl sample of digested DNA (~50 ng) was run on 1% agarose gel to verify the effectiveness of digestion. A total of 20 µl of ligation master mix consisted of 0.2 µM of each specific adaptor (five pairs of adaptors per five samples), 0.5 mM ATP (New England Biolabs), 200 U T4 DNA ligase (New England Biolabs), 2 µl 10 × T4 ligase buffer, 5.9 µl nuclease‐free water, and 10 µl digestion product. Each reaction tube was incubated at 16°C for 1 hr. The ligation products were amplified in 50 µl PCRs. Each sample was composed of 0.16 µM of each primer, 0.24 mM dNTP, 10 µl 5 × HF buffer, 0.8 U Phusion high‐fidelity DNA polymerase (New England Biolabs), 18.8 µl nuclease‐free water, and 18 µl ligation products. PCR was conducted in MyCycler thermal cyclers (Bio‐Rad) with 16 cycles of 98°C for 5 s, 60°C for 20 s, and 72°C for 10 s. Fifty microliter of each PCR product was run on an 8% polyacrylamide gel, with the DNA diffused into nuclease‐free water at 37°C for 30 min. For each tube, 12 µl of supernatant was used as a template and the above PCR steps repeated for 4–6 PCR cycles to improve the yield. The PCR products from five samples were combined, and the mixture purified using a MinElute PCR Purification Kit. Thirty microliter of digestion master mix was prepared containing 1 mM ATP, 3 µl 10 × CutSmart buffer, 2 U SapI (New England Biolabs), 10 µl purified mixed PCR product, and 13.8 µl nuclease‐free water. The mixture was then incubated at 37°C for 30 min. The digested product was added to the tube containing pretreated magnetic beads and the mixture incubated at room temperature. After a magnet was applied and the supernatant transferred to a new tube, 200 U T4 DNA ligase was added to the supernatant and incubated at 16°C for 45 min. Gel purification was then performed as described above. Barcodes were introduced by PCR with barcode‐bearing primers. PCR products were purified using a MinElute PCR Purification Kit and pooled for sequencing using the Illumina Hiseq Xten Paired‐end sequencing platform.

### 2.4 Data filtering and SNP identification

We filtered the raw sequence data as follows: first, the reads with linker sequences were removed to obtain clean reads; second, reads with low‐quality positions (>15% of nucleotide positions with a Phred score <30) were deleted. In *N* bases >8% and without restriction recognition sites, the filtered high‐quality sequences are referred to as enzyme reads. Then, mapping the enzyme reads to the *B. tabaci* MEAM1 and MED reference genomes using SOAP program (the parameter was set to ‐r0‐M4‐v2; Chen et al., 2016; Li et al., 2009; Xie et al., 2017) and the same reads cluster into Unique Tags. Finally, the SNP‐calling was performed using Maximum likelihood (ML) method (Fu et al., 2013). In order to ensure the accuracy of SNP genotyping, the following filtering procedures were performed: (a) SNPs with a minor allele frequency (MAF) <0.01 were deleted; (b) tags with more than 2 SNPs were deleted; (c) SNPs at each locus with 1 or 4 bases were deleted; (d) SNPs that could be genotyped in more than 80% of the individuals were retained.

### 2.5 Evaluating the effects of sample size

Resampling techniques and an ad hoc statistic, Δ*K*, were used to identify the effect sample size has on estimates of genetic diversity and differentiation. Prior to evaluating the optimal sample size directly, we conducted a power analysis to find out the minimum number of resampling replicates that would be demanded to ensure exact estimation of genetic parameters. For each population, we first randomly selected 3,000 SNPs using data tools in Excel, we then constructed simulated data sets comprised of different numbers of resampling replicates (*x* = 10, 20, 30, 40, 50, 60, 70, 80, 90, 100) each represented by all combination of the different sample sizes (*n* = 2, 3, 4, 5, 6, 7, 8). To construct each simulated population, a macro in Excel designed to assign each individual in the empirical data set a random number (between 1 and 10,000) was used to select a random subset of individuals from the empirical data set (*n* = 10). After sorting the data set by the random numbers, then we assigned the first two (or three, four, etc. depending on the sample size category) to a new worksheet multiplied by 10 (or 20, 30, etc. depending on the replicates category), bringing about simulated “populations” that were random, independent subsamples of the empirical data set. Sampling was carried out without alternate, so in the same replicate no *B. tabaci* individual was included more than once (just as in a realistic population genetic data set). Since replicates were independent of each other, however, the same individual may be contained in more than one replicate of the simulated data set. GenALEx 6.5 was used to calculate unbiased expected heterozygosity (u*H*_e_), observed heterozygosity (*H*_o_), the number of effect alleles (*A*_e_), and pairwise genetic differentiation (*F*_ST_) for each replicate at each sample size (Peakall & Smouse, 2006). The biotype identification results showed that SPB and 17JN populations were identified as Q1 clade while USA0601 and ISQ populations were Q2 clade. Research has provided that *B. tabaci* subclade Q1 in Jinan originated in the western Mediterranean (e.g., Spain) and *B. tabaci* subclade Q2 in USA likely originated from the Israel (Chu, Gao, De Barro, Wan, & Zhang, 2011; Chu et al., 2008). So, we calculated the *F*_ST_ of Q1 and Q2 subclades, respectively.

We used box plots to measure the influence of sample sizes and replicates on intra‐ and interpopulation genetic diversity parameters. Box plots are based on statistics that do not require an assumption regarding the shape of the data distribution (Krzywinski & Altman, 2014). To judge the differences between means, 95% confidence intervals were used and were introduced in the box plots using BOXPLOTR (Spitzer, Wildenhain, Rappsilber, & Tyers, 2014).

An ad hoc statistic, Δ*K*, was used to assist in judging the optimal replicates and sample sizes for population genomics (Evanno et al., 2005). We used the height of the Δ*K* as an indicator of the optimal number.

## 3 RESULTS

### 3.1 Biotype identification and 2b‐RAD data matrix analysis

**Figure 1:**
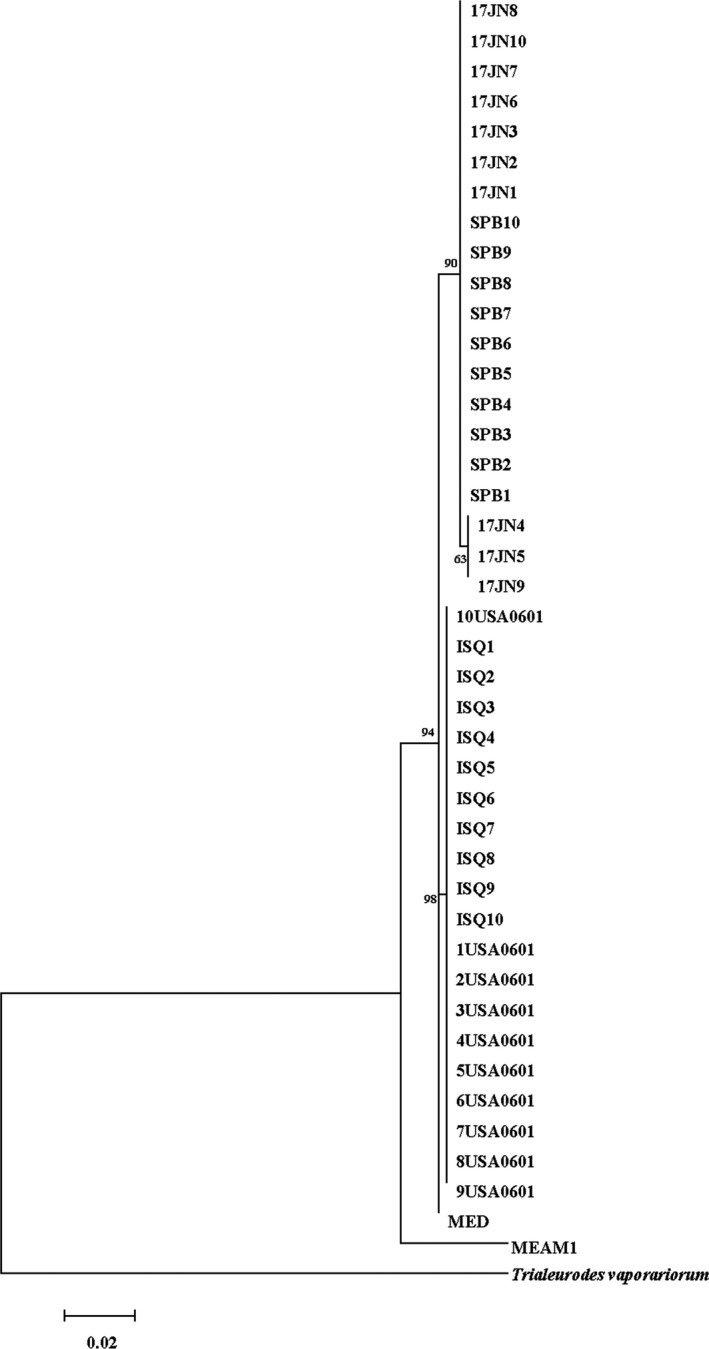
Ml tree for mtCOI sequences of *Bemisia tabaci* MED and sequences presented in our study. The outgroup was mtCOI sequences of *Trialeurofes vaporariorum*

Based on the 482 bp mtCOI sequences, the genetic distance of all individuals between the subclades of *B. tabaci* Q was 0.00 and the distance between *B. tabaci* Q and *T. vaporariorum* was 0.27. The ML trees (Figure 1) revealed a similar result confirming that all individuals were *B. tabaci* Q. Of the 40 whitefly samples, a total of two clades (Q1 and Q2) were identified. SPB and 17JN populations were identified as Q1 clade while USA0601 and ISQ populations were Q2 clade (Figure 1).

A total of 40 individuals were used for the next sequencing. Sequencing of 40 individuals generated 211,661,317 clean reads, with an average of 5,291,533 clean reads per sample. After deleting the low‐quality reads, a total of 142,621,503 enzyme reads were obtained (67.38% of the clean reads). These reads were aligned to the *B. tabaci* MEAM1 and MED genomes (Chen et al., 2016; Xie et al., 2017). An average of 94,386 unique tags were aligned to the genome sequence with an average depth of 19. Finally, data sets of 7,867, 7,460, 13,068, and 16,606 polymorphic SNPs for the ISQ, USA0601, SPB, and 17JN populations, respectively, passed the default filters and were used for the following study. The number of effect alleles (*A*_e_) in the *B. tabaci* populations ranged from 1.179 (SPB) to 1.103 (USA0601). The expected heterozygosity (*He*) in the *B. tabaci* populations ranged from 0.063 (USA0601) to 0.109 (SPB). The observed heterozygosity (*H*_o_) in the four populations ranged from 0.059 (ISQ) to 0.106 (17JN). The *F*_ST_ distance of SPB and 17JN calculated from all SNPs was 0.0778. The *F*_ST_ distance of USA0601 and ISQ calculated from all SNPs was 0.3457, indicating that a substantial genetic differentiation exists between the two populations.

### 3.2 Determination of the sample sizes for intrapopulation genetic diversity

**Figure 2:**
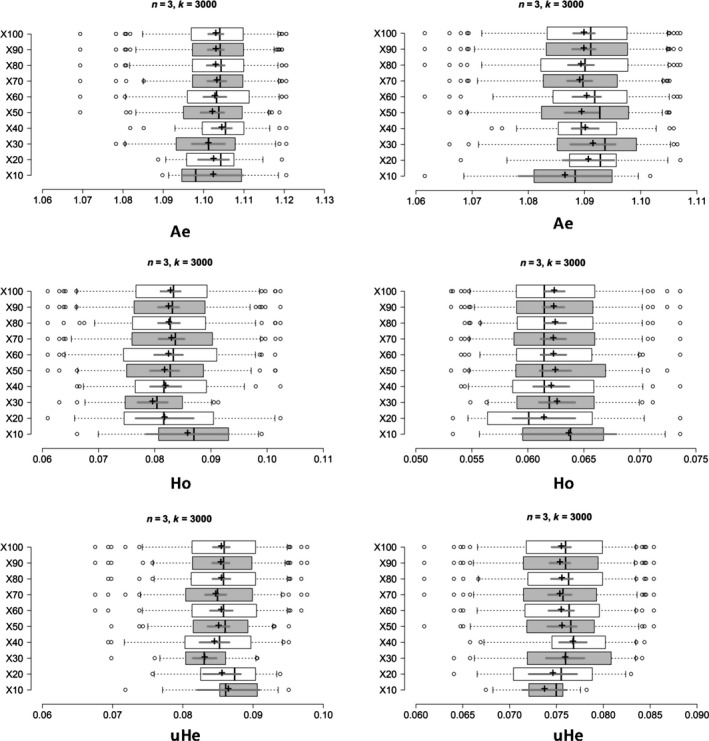
Boxplots showing the minimum number of resampling replicates (*x*) needed to obtain accurate estimates of genetic diversity for populations of *Bemisia tabaci* (USA0601 and ISQ), the boxplots on the left and right represent the results of USA0601 and ISQ separately. Center lines show the medians; box limits indicate the 25th and 75th percentiles; whiskers extend 1.5 times the interquartile range from the 25th and 75th percentiles; outliers are represented by dots; crosses represent sample means; bars indicate 95% confidence intervals of the means. *X* from 10 to 100 resampling replicates is the sample point. *A*_e_, number of effective alleles; *H*_o_, observed heterozygosity; u*H*_e_, unbiased expected heterozygosity

**Figure 3:**
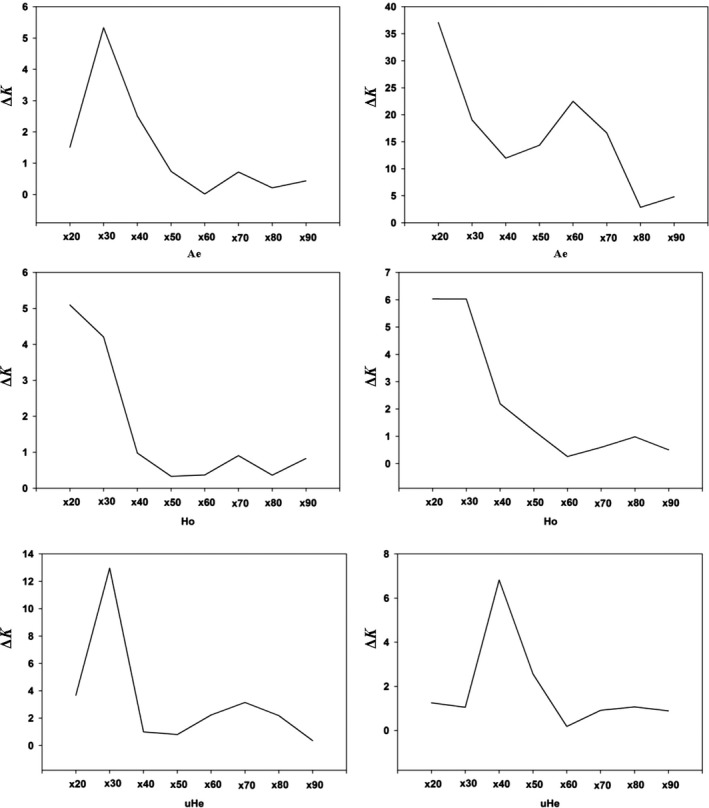
Line charts showing the minimum number of resampling replicates (*x*) needed to obtain accurate estimates of genetic diversity for populations of *Bemisia tabaci* (USA0601 and ISQ), the line charts on the left and right represent the results of USA0601 and ISQ separately. The Δ*K* (*y*‐axis) showed a peak at the optimal replicates (*x*). *A*_e_, number of effective alleles; *H*_o_, observed heterozygosity; u*H*_e_, unbiased expected heterozygosity

We evaluated the influence of rising sample sizes for intra‐ and interpopulation genetic diversity valuation by resampling 2 to 8 samples from empirical data sets attained for the four *B. tabaci* populations. Precise estimates of population genetic parameters were obtained in our simulations with *x* = 20, 30 or 40 resampling replicates (Figure 2 and Figure S1). Using the ISQ population as an example, when we fixed the number of samples to three (*n* = 3) and the number of SNPs to 3,000, we detected no statistical difference for the mean values of *A*_e_, *H*_o_ and u*H*_e_ while the number of replicates was set to *x* = 20, 20, and 40, respectively (*A*_e_ = 1.091, 95% CI [1.086, 1.095]; *H*_o_ = 0.061, 95% CI [0.059, 0.064]; and u*H*_e_ = 0.077, 95% CI [0.075, 0.078]) or *x* = 100 (*A*_e_ = 1.090, 95% CI [1.089, 1.092]; *H*_o_ = 0.062, 95% CI [0.061, 0.063]; and u*H*_e_ = 0.076, 95% CI [0.074, 0.077]). Concurrently, the Δ*K* line chart shows a peak at *x* = 20, 20, and 40 separately (Figure 3). For the other three populations, precise estimates of population genetic parameters (*A*_e_, *H*_o_, and u*H*_e_) were obtained in our simulations with *x* = 20, 30, or 40 resampling replicates (Figure 3 and Figure S2).

**Figure 4:**
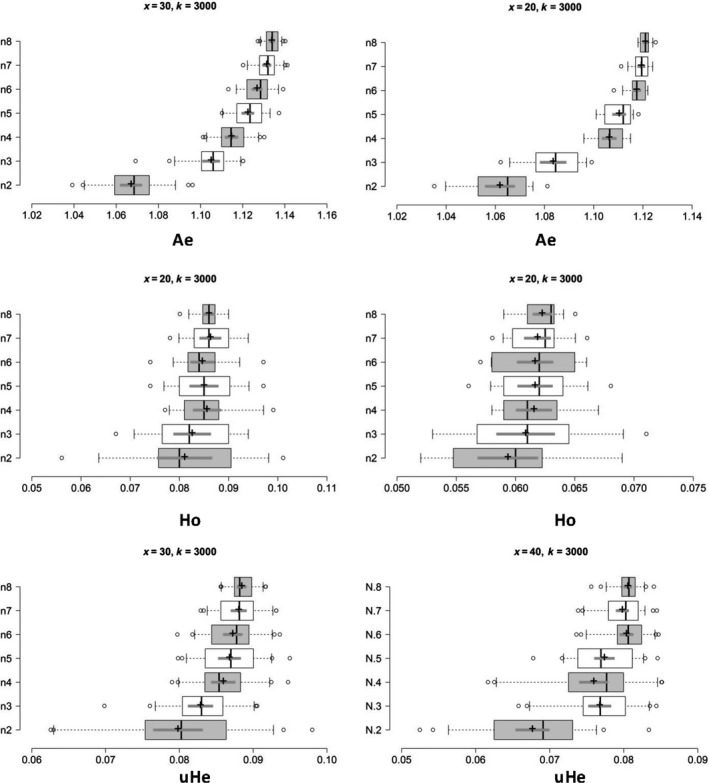
Based on the optimal replicates (*x*) for populations of *Bemisia tabaci*, boxplots showing the minimum number of sample sizes (*n*) needed to obtain accurate estimates of genetic diversity for populations of *Bemisia tabaci* (USA0601 and ISQ), the boxplots on the left and right represent the results of USA0601 and ISQ separately. Center lines show the medians; box limits indicate the 25th and 75th percentiles; whiskers extend 1.5 times the interquartile range from the 25th and 75th percentiles; outliers are represented by dots; crosses represent sample means; bars indicate 95% confidence intervals of the means. *n* is the sample size. *A*_e_, number of effective alleles; *H*_o_, observed heterozygosity; u*H*_e_, unbiased expected heterozygosity

**Figure 5:**
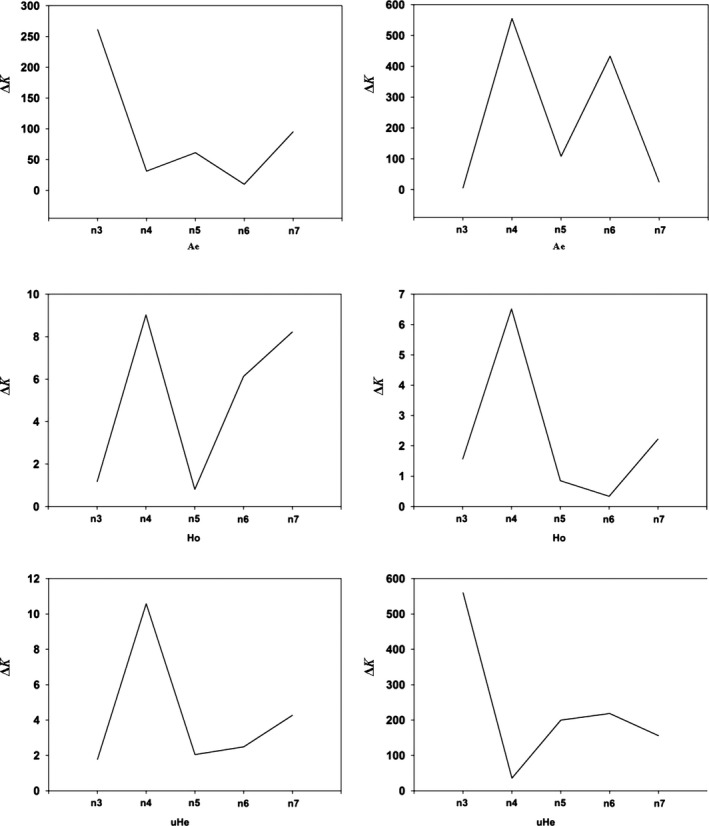
Line charts showing the minimum number of sample sizes (*n*) needed to obtain accurate estimates of genetic diversity for populations of *Bemisia tabaci* (USA0601 and ISQ), the line charts on the left and right represent the results of USA0601 and ISQ separately. The Δ*K* (*y*‐axis) showed a peak at the minimum sample sizes (*n*). *A*_e_, number of effective alleles; *H*_o_, observed heterozygosity; u*H*_e_, unbiased expected heterozygosity

Our simulations were able to determine the minimum sample size of *B. tabaci* required to make sure that the sample precisely reflects the genetic diversity of the empirical data sets. In the ISQ population, raising sample sizes above three (*n* ≥ 3) individuals appears to have slight impact on the mean u*H*_e_. Such as, the mean value of unbiased *He* for *n* = 3 was 0.077 (95% CI [0.075, 0.078]) and for *n* = 8 it was 0.080 (95% CI [0.080, 0.081]) (Figure 4). At the same time, the Δ*K* line chart shows a clear peak at *n* = 3 (Figure 5). In the *A*_e_ and *H*_o_ estimates, a small sample size (*n* = 4) with 3,000 SNPs was adequate to accurately reflect the genetic diversity found in the ISQ population (Figure 4); the mean value and 95% CI were listed in Table 1.

**Table 1:** The mean and 95% CI for different genetic parameters

Populations	Genetic diversity						Genetic differentiation	
	*A*_e_		*H*_o_		u*H*_e_		*F*_ST_	
	Mean	95% CI	Mean	95% CI	Mean	95% CI	Mean	95% CI
ISQ	1.106 (*n* = 4)	1.103, 1.109	0.061 (*n* = 4)	0.060, 0.063	0.077 (*n* = 3)	0.075, 0.078	0.231 (*n* = 3)	0.223, 0.234
	1.121 (*n* = 8)	1.120, 1.122	0.062 (*n* = 8)	0.061, 0.063	0.080 (*n* = 8)	0.080, 0.081		
USA0601	1.134 (*n* = 3)	1.133, 1.135	0.086 (*n* = 4)	0.083, 0.088	0.056 (*n* = 4)	0.054, 0.058	0.242 (*n* = 8)	0.239, 0.244
	1.105 (*n* = 8)	1.101, 1.109	0.086 (*n* = 8)	0.085, 0.087	0.059 (*n* = 8)	0.058, 0.060		
17JN	1.162 (*n* = 3)	1.158, 1.166	0.125 (*n* = 4)	0.122, 0.128	0.121 (*n* = 3)	0.119, 0.124	0.056 (*n* = 4)	0.052, 0.060
	1.175 (*n* = 8)	1.174, 1.176	0.121 (*n* = 8)	0.119, 0.122	0.120 (*n* = 8)	0.120, 0.121		
SPB	1.131 (*n* = 3)	1.127, 1.135	0.0101 (*n* = 3)	0.0098, 0.0104	0.092 (*n* = 3)	0.090, 0.094	0.070 (*n* = 8)	0.067, 0.073
	1.166 (*n* = 8)	1.165, 1.167	0.029 (*n* = 8)	0.027, 0.031	0.106 (*n* = 8)	0.106, 0.107		

In the USA0601 population, sample sizes more than four individuals appear to have only a trivial influence on the mean u*H*_e_ when using 3,000 SNPs. The mean values of unbiased *He* for *n* = 4 was 0.056 (95% CI [0.054, 0.058]), and for *n* = 8, it was 0.059 (95% CI [0.058, 0.060]). Simultaneously, the Δ*K* line chart shows a clear peak at *n* = 4 (Figure 5). Moreover, small sample sizes (*n* = 3 or 4) were enough to recover the *A*_e_ and *H*_o_ for the USA0601 population (Figure 4). The Δ*K* line chart also shows peaks at *n* = 3 and 4 during this time (Figure 5).

In the 17JN population, sample size higher than three individuals seems to have a negligible effect on the mean u*H*_e_ when 3,000 SNPs are considered. The mean values of unbiased *He* for *n* = 3 was 0.121 (95% CI [0.119, 0.124]), and for *n* = 8, it was 0.120 (95% CI [0.120, 0.121]) (Figure S3). Simultaneously, the Δ*K* line chart shows a clear peak at *n* = 3 (Figure S4). Moreover, small sample sizes (*n* = 3 or 4) were enough to recover the *A*_e_ and *H*_o_ for the 17JN population (Figure S3). The Δ*K* line chart also shows peaks at *n* = 3 and 4 during this time (Figure S4). Furthermore, to reduce the overlaps between subsampled replicates, we constructed simulated data sets comprised of different numbers of sample sizes (*n* = 2–6) and using 1,000 SNP. The results showed that a sample size greater than four individuals has little impact on estimates of genetic diversity within JN population (Figure S7).

In the SPB population, sample sizes more than three individuals seem to have only a trivial effect on the mean u*H*_e_ when 3,000 SNPs are used. The mean value of unbiased *He* for *n* = 3 was 0.092 (95% CI [0.090, 0.094]), and for *n* = 8, it was 0.106 (95% CI [0.106, 0.107]). Simultaneously, the Δ*K* line chart shows a peak at *n* = 3 (Figure S4). Moreover, small sample sizes (*n* = 3) were enough to recover the *A*_e_ and *H*_o_ for the SPB population (Figure S3). The Δ*K* line chart also shows peaks at *n* = 3 during this time (Figure S4).

### 3.3 Determination of the sample sizes for interpopulation genetic diversity

**Figure 6:**
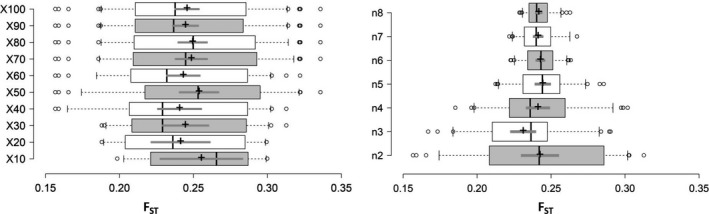
Boxplots showing the optimal replicates (left) and minimum number of sample sizes (right) needed to obtain accurate estimates of *F*_ST_ between populations of *Bemisia tabaci* (USA0601 and ISQ). Center lines show the medians; box limits indicate the 25th and 75th percentiles; whiskers extend 1.5 times the interquartile range from the 25th and 75th percentiles; outliers are represented by dots; crosses represent sample means; bars indicate 95% confidence intervals of the means. *n*, sample sizes; *x*, resampling replicates

**Figure 7:**
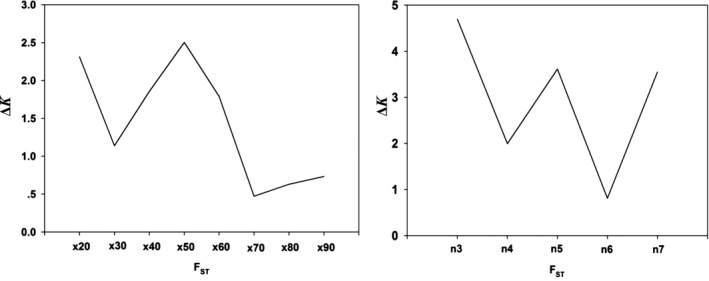
Line charts showing the optimal replicates (left) and minimum number of sample sizes (right) needed to obtain accurate estimates of *F*_ST_ between populations of *Bemisia tabaci* (USA0601 and ISQ). The Δ*K* (*y*‐axis) showed a peak at the optimal replicates and sample sizes. *x*, resampling replicates; *n*, sample sizes

In terms of the degree of genetic differentiation, for the populations of USA0601 and ISQ, when the number of individuals (*n*) was fixed to three and the number of SNPs were fixed to 3,000. The result showed that compared with *x* = 100, no statistical difference was tested for the mean values of *F*_ST_ when we set the number of replicates to *x* = 50 (Figure 6). For example, the mean values of *F*_ST_ for *x* = 50 were 0.254 (95% CI [0.240, 0.267]) and for *x* = 100 were 0.246 (95% CI [0.237, 0.254]). At the same time, the Δ*K* line chart showed a peak at *x* = 50 (Figure 7). Based on 50 replicates, it seems that the impact on the mean *F*_ST_ is slight when raising sample size above three (Figure 6). Furthermore, the Δ*K* line chart shows a peak at *n* = 3 (Figure 7).

For the populations of 17JN and SPB, when we set the number of individuals (*n*) to three and the number of SNPs to 3,000, compared with *x* = 100, we detected no statistical difference for the mean values of *F*_ST_ when the quantity of replicates was set to *x* = 30 (Figure S5). For example, the mean values of *F*_ST_ for *x* = 30 were 0.106 (95% CI [0.091, 0.122]) and for *x* = 100 were 0.091 (95% CI [0.083, 0.099]). At the same time, the Δ*K* line chart showed a clear peak at *x* = 30 (Figure S6). Based on 30 replicates, increasing sample size above four individuals seems to have slight impact on the mean *F*_ST_ (Figure S5). Furthermore, the Δ*K* line chart shows a clear peak at *n* = 4 (Figure S6).

## 4 DISCUSSION

Previous studies have demonstrated that invasion genetics can be affected by many factors including the bottleneck effect, founder effect, bridgehead effect, multiple introductions, genetic turnover, gene flow, and hybridization (Chu, Qu, & Guo, 2018). Considering the potential effects of these factors, it is imperative to analyze the sampling scheme when studying population genomics of invasive species.

Genome‐wide high‐throughput techniques based on RADseq have been widely used in ascertaining the population genomics of invasive species (Elfekih et al., 2018; Resh et al., 2018; Yi et al., 2018). Nevertheless, the choice of the sample sizes for such studies is generally ad hoc, and related studies are still scarce (Hoban, Gaggiotti, & Bertorelle, 2013). Our study is the first to use empirical data to find out the sample sizes needed for accurate estimates of population genomics of invasive species. Our results showed that even with a limited number of individuals, accurate estimations of genetic diversity and differentiation can be obtained by using RADseq that provide a large number of SNPs, which is similar but not the same as the conclusion based on simulation (Willing et al., 2012) or empirical analysis (Nazareno et al., 2017). For example, Nazareno et al. (2017) stated that the accurate genetic diversity could be obtained using only six to eight individuals when a large number of SNP loci were used, whereas our present study determined that only three to four individuals could be required to recover within‐population genetic diversity parameters. There may be two reasons for this difference; on the one hand, we noted that the minimum sample size for insects (whiteflies in the present study) is smaller than that required for analyzing plants (six to eight individuals; Nazareno et al., 2017), which may be closely associated with the high vagility of most winged insects—being instrumental in promoting gene flow between individuals and populations. On the other hand, the sampling space in the data set in our study (*n* = 10) is smaller than Nazareno et al. (*n* = 35), too limited sampling space would increase overlaps between iterations. A similar study evaluated 30 individuals each population to identify the optimal sample size (Flesch, Rotella, Thomson, Graves, & Garrott, 2018), and a previous simulation study also recommended that 25–30 samples should be used per population (Hoban & Schlarbaum, 2014). To generate more unique replicates, a greater number of samples (>30) should be used in further studies. To reduce the overlaps between subsampled replicates, based on the data set we have, we constructed simulated data sets comprised of different numbers of sample sizes (*n* = 2–6) and the results showed that a sample size greater than four individuals has little impact on estimates of genetic diversity within JN population. The results demonstrated that our conclusion was not an artifact.

In addition, our study also revealed that the genetic diversity of a given population can, in most cases, be accurately reflected using small sample sizes, although these may vary by region. For example, the minimum sample sizes required for the diversity parameter of the introduced whitefly population from the USA differ from those needed for the native whitefly population in Israel. Similarly, the minimum sample sizes required for diversity parameter of the introduced whitefly population from China (Jinan) differ from those needed for the native whitefly population in Spain. This may be associated with the effects of invasion being coupling with bottleneck effects, founding effects, etc.

Compared with traditional molecular markers, the finer population differentiation can be obtained with lower sample sizes when using genome‐wide high‐throughput techniques (Jeffries et al., 2016; Willing et al., 2012). Here, we identified the number of individuals needed to recover population differentiation of invasive species with a large number of SNPs. Our results demonstrated that even when sample sizes are small (*n* = 3 or 4), unbiased estimates of population differentiation can still be obtained (Figure 7 and Figure S5). However, the minimum sample size in our study is lower than that needed for simulation (four to six individuals; Willing et al., 2012) but higher than in empirical analysis (only two individuals; Nazareno et al., 2017; Table 2). These results have shown that the actual minimum sample size necessary when studying genetic differentiation of invasive species is lower than simulation analysis. It has also shown that by analysis of the invasive whitefly, *B. tabaci* MED, the actual minimum sample size needed for invasive genomics is not dramatically affected by the invasion process.

**Table 2:** The optimal sample sizes required for different genetic parameters

Species analyzed	Genetic diversity			Genetic differentiation (*F*_ST_)	References
	*A*_e_	*H*_o_	u*H*_e_		
*Bemisia tabaci* MED Q1 clade	3	3–4	3	4	The present study
*Bemisia tabaci* MED Q2 clade	3–4	4	3–4	3	The present study
*Amphirrhox longifolia*	2	2	6–8	2	Nazareno et al. (2017)

The population genetics of *B. tabaci* has been studied using multiple molecular marker methods such as microsatellite loci (Chu et al., 2011, 2013) or RAD sequencing (Elfekih et al., 2018). For example, the population genetics of *B. tabaci* using the RAD sequencing method by Elfekih et al. (2018) did not attempt to evaluate the minimum sample size, in which the sample sizes ranged from two to nine individuals. Since *B. tabaci* is a species complex which includes numerous cryptic species (De Barro, 2012), the potential effects that the different cryptic taxa may have on the sample size should be explored further. In addition, our study was limited to a small number of SNPs (3,000) and limited sampling space. A similar study used 23,057 SNPs to determine optimal sample size of *Galapagos tortoise* (Gaughran et al., 2017). Another empirical simulation study employed approximately 14,000 SNPs to define optimal sampling strategies for free‐ranging mammals (Flesch et al., 2018). In recent years, genomics was extensively utilized to study population genetic and diversity of invasive species (Yi et al., 2018), and the sample size selection is per se a crucial issue in the analysis. Thus, in further study, more individuals and SNPs should be used in evaluating optimal sample size of invasion spaces.

Our studies suggest that a relatively small sample size can be used for an accurate estimation of genetic diversity and differentiation of alien species, which will help to provide a foundation for future research on the population genomics of invasive species. The necessity of resorting to small sample sizes in invasion genomics is helpful in revealing the invasion genetics of alien species, especially since most invasions consist of limited sized populations during their initial colonization and establishment phases (Lockwood, Hoopes, & Marchetti, 2007). However, alternative molecular marker methods may require many individuals. For example, 25–30 individuals per population are often needed to accurately estimate the genetic diversity using microsatellite‐based population genetic studies (Hale et al., 2012).

## CONFLICT OF INTEREST

None declared.

## AUTHOR CONTRIBUTIONS

DC conceived the ideas and designed the study; ZKW and YGZ provided the data analysis methods; WMQ and NL analyzed the data and wrote the manuscript; all authors edited the manuscript.

## Data Availability

Raw data: NCBI BioProject PRJNA516276. Raw sequences: SRR8468886‐SRR8468925. Data archiving: Supplementary information is available at Ecology and Evolution's website.
