# Supplementary figures and images for: Minimum sample sizes for invasion genomics: Empirical investigation in an invasive whitefly
Source: Ecol Evol. 2019 Oct 2;10(1):38–49. doi: 10.1002/ece3.5677 (PMC6972819; doi:10.1002/ece3.5677)

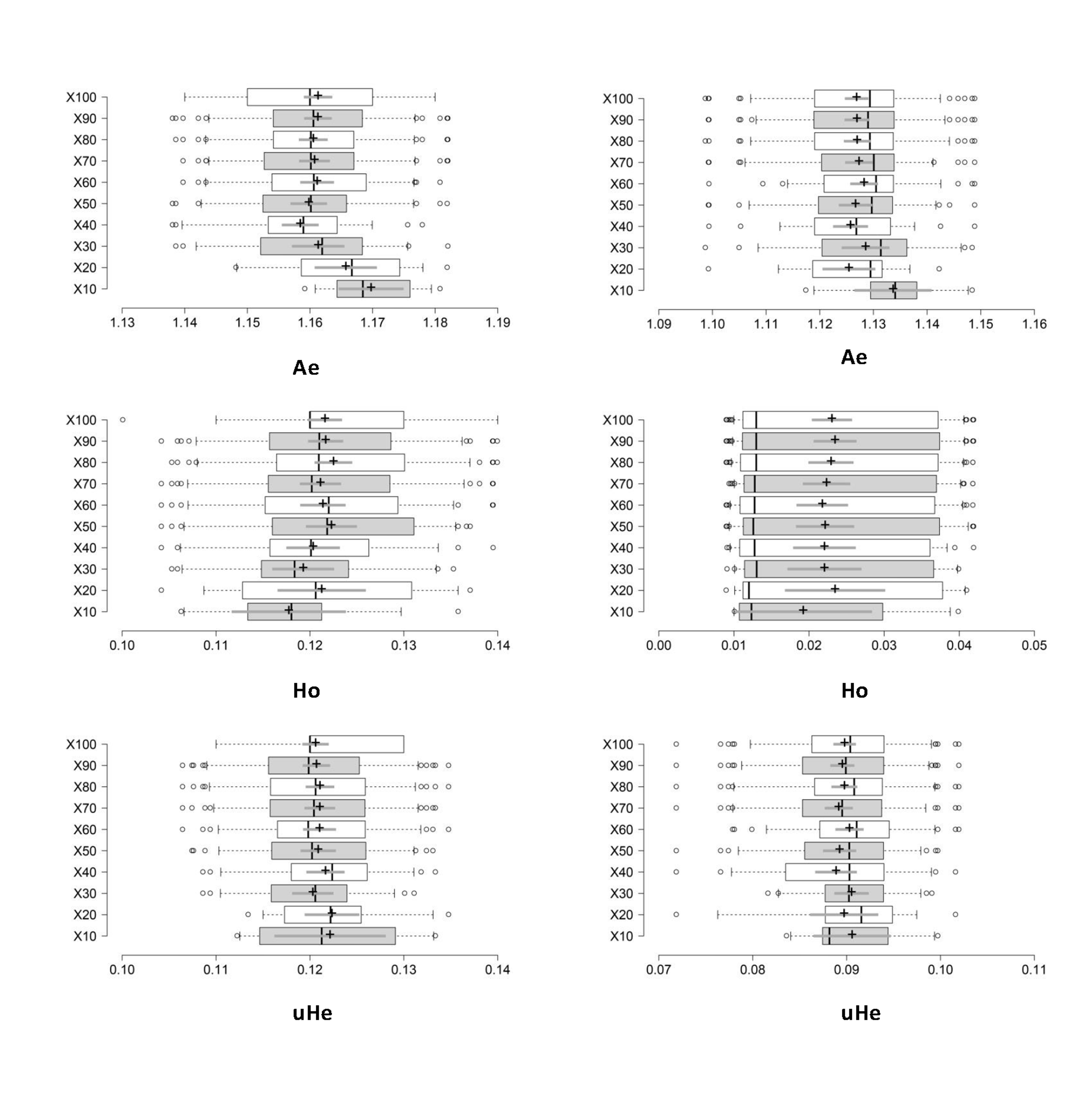

Supplement: Supplementary file 1 [file ECE3-10-38-s001.png]

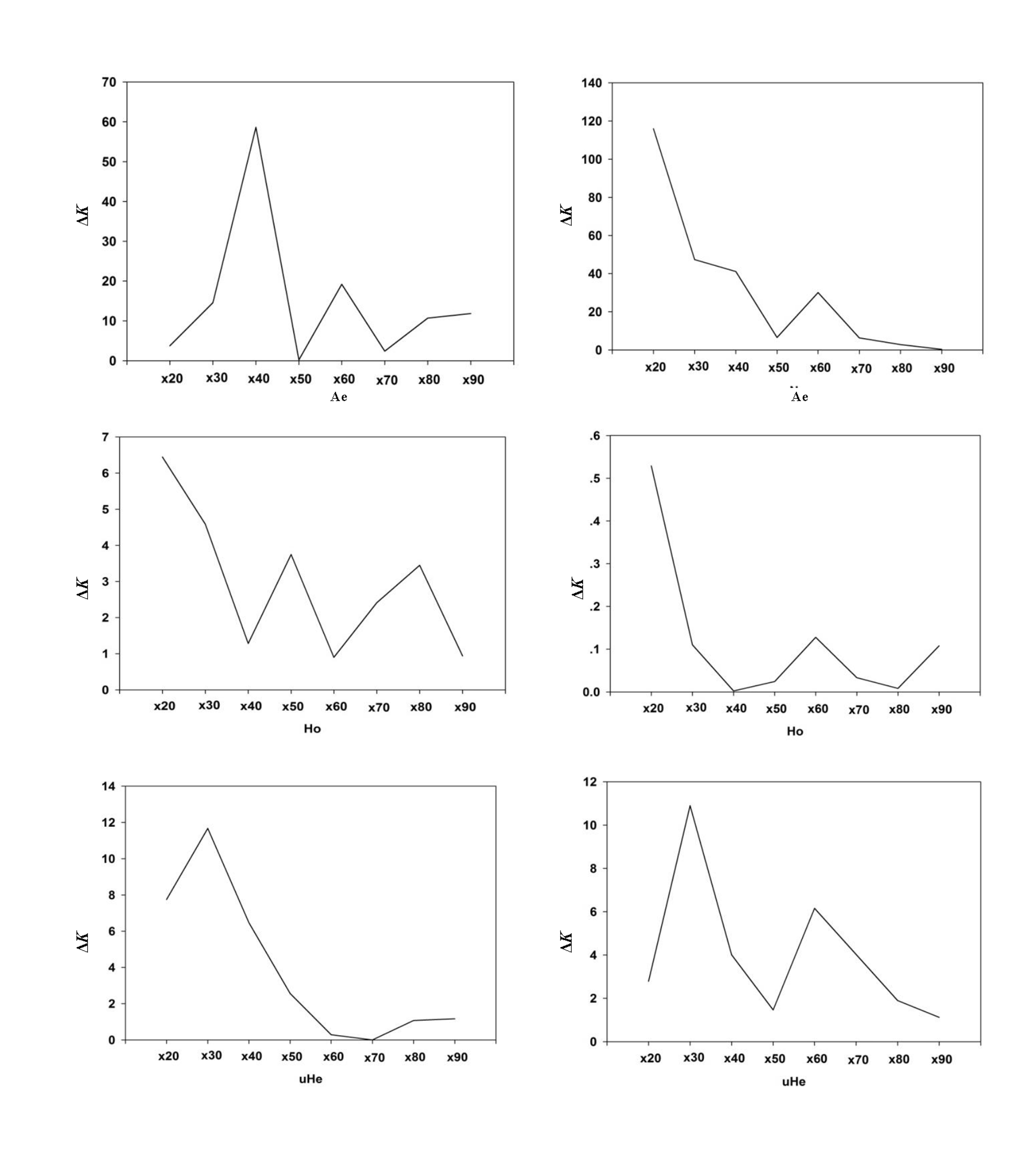

Supplement: Supplementary file 2 [file ECE3-10-38-s002.png]

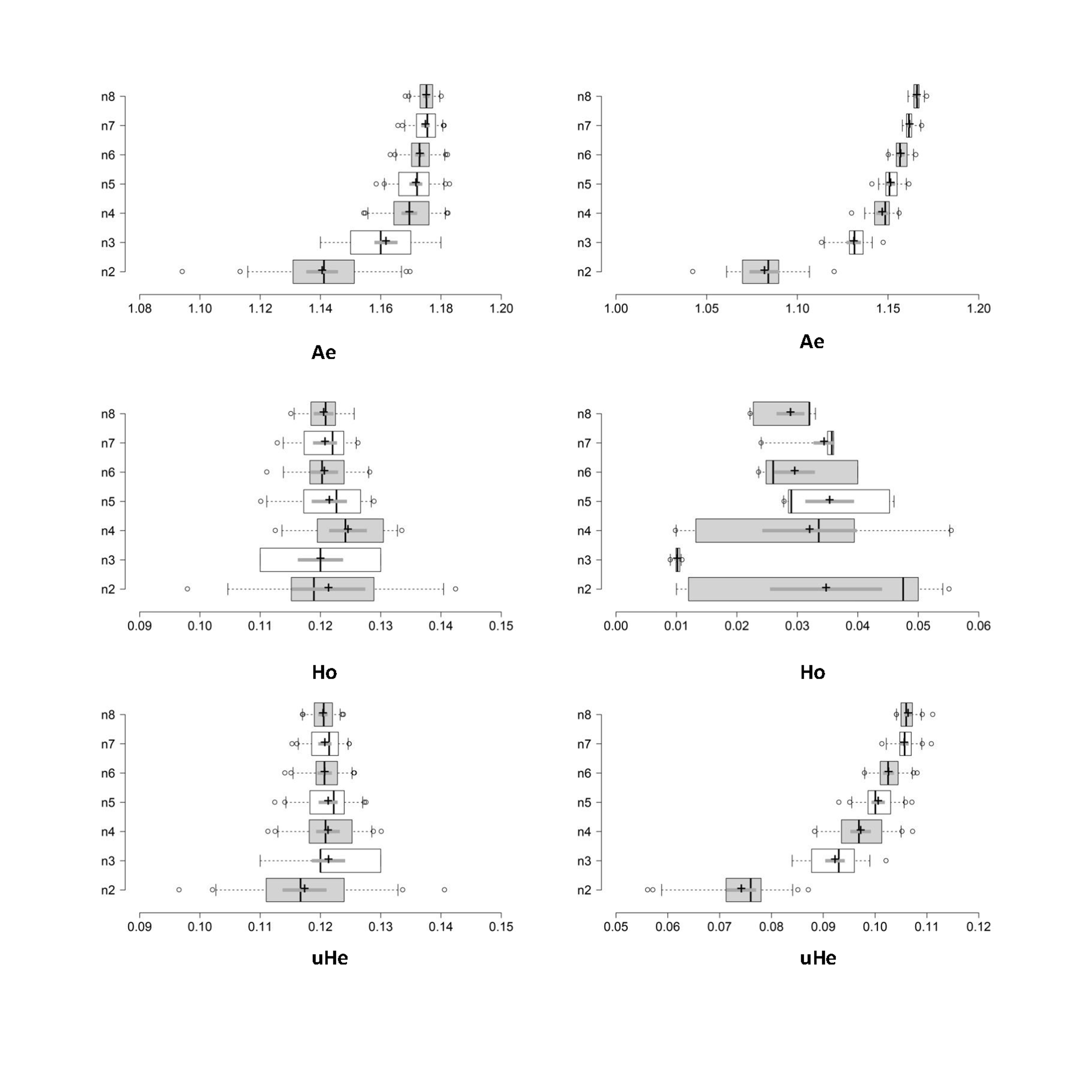

Supplement: Supplementary file 3 [file ECE3-10-38-s003.png]

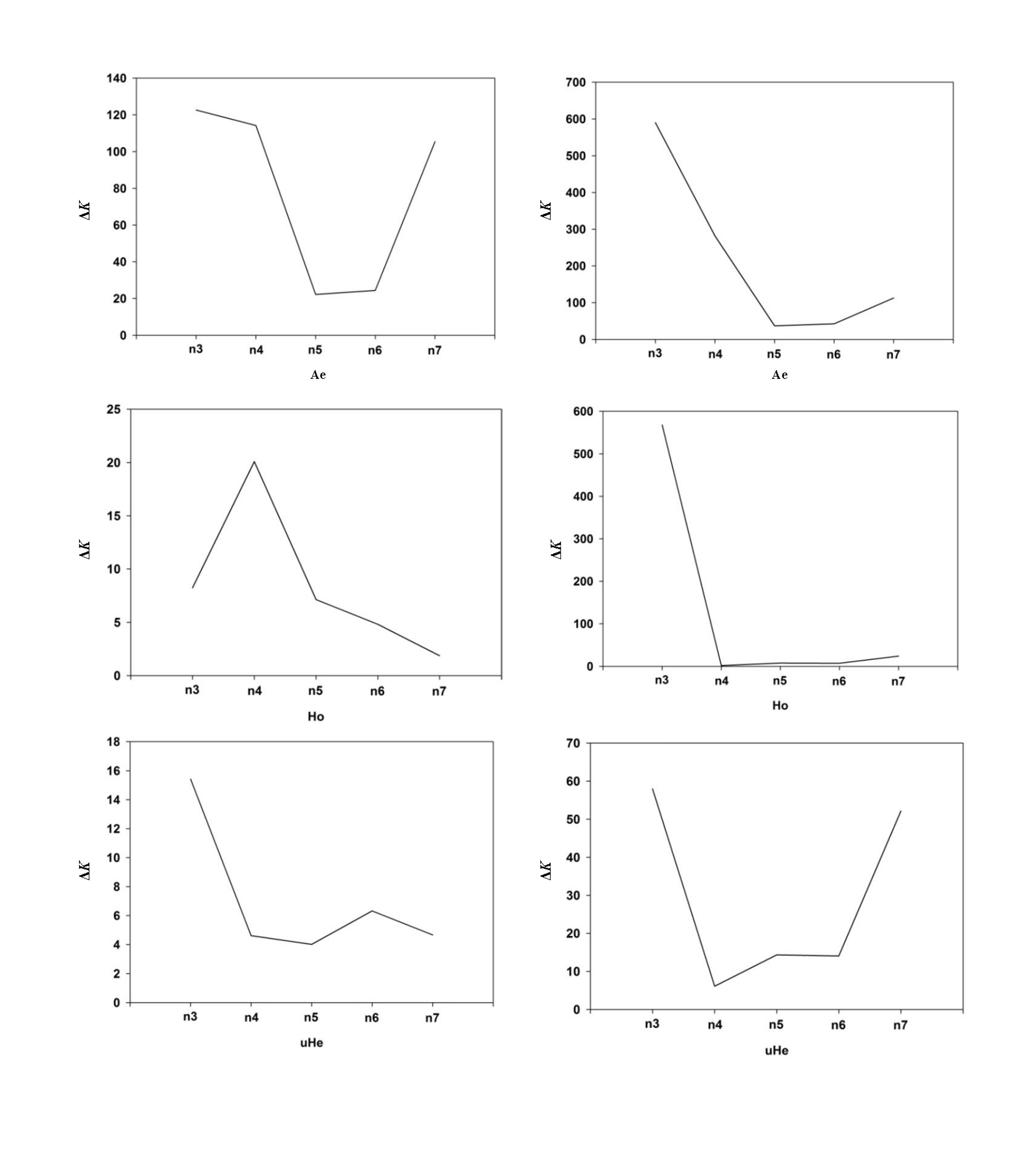

Supplement: Supplementary file 4 [file ECE3-10-38-s004.png]

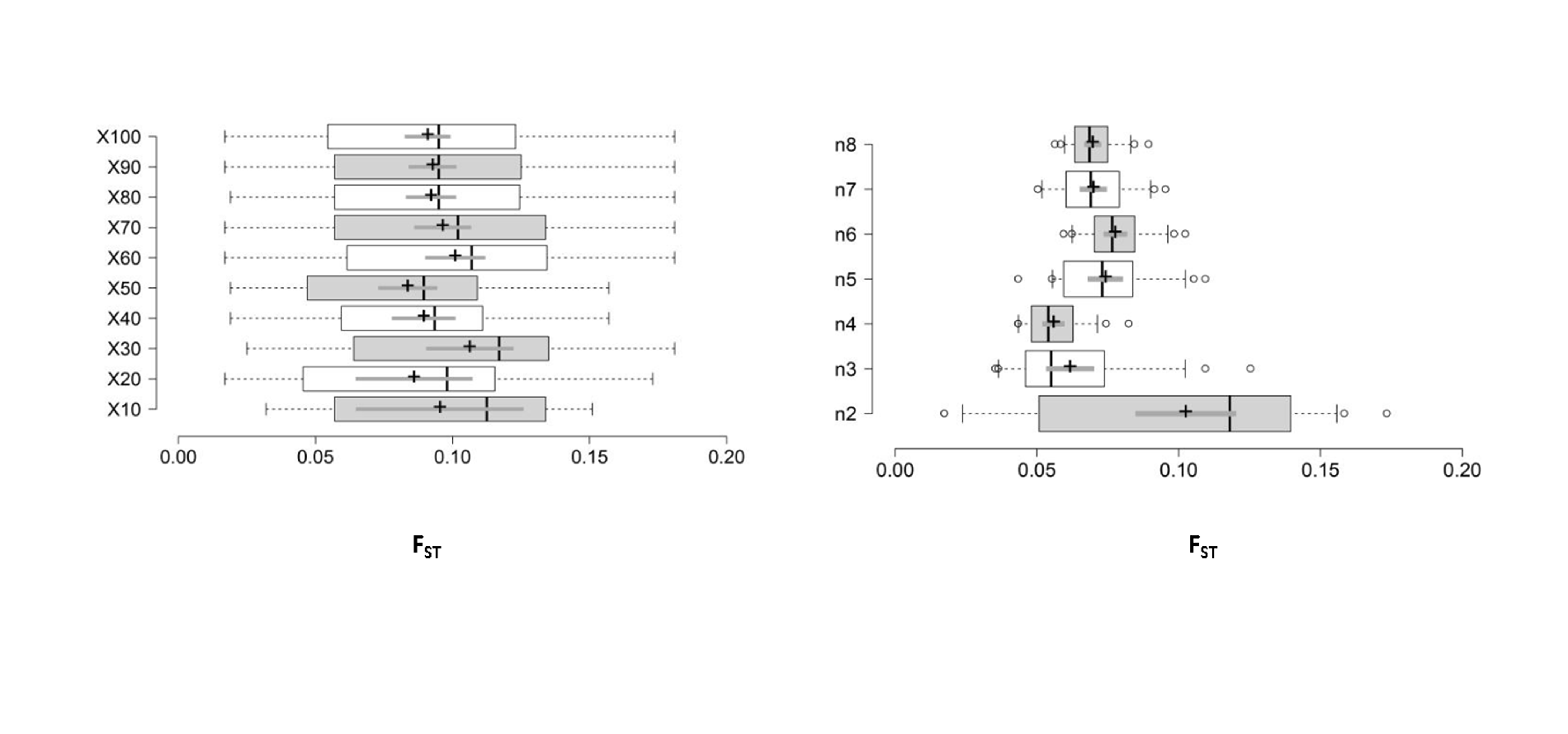

Supplement: Supplementary file 5 [file ECE3-10-38-s005.png]

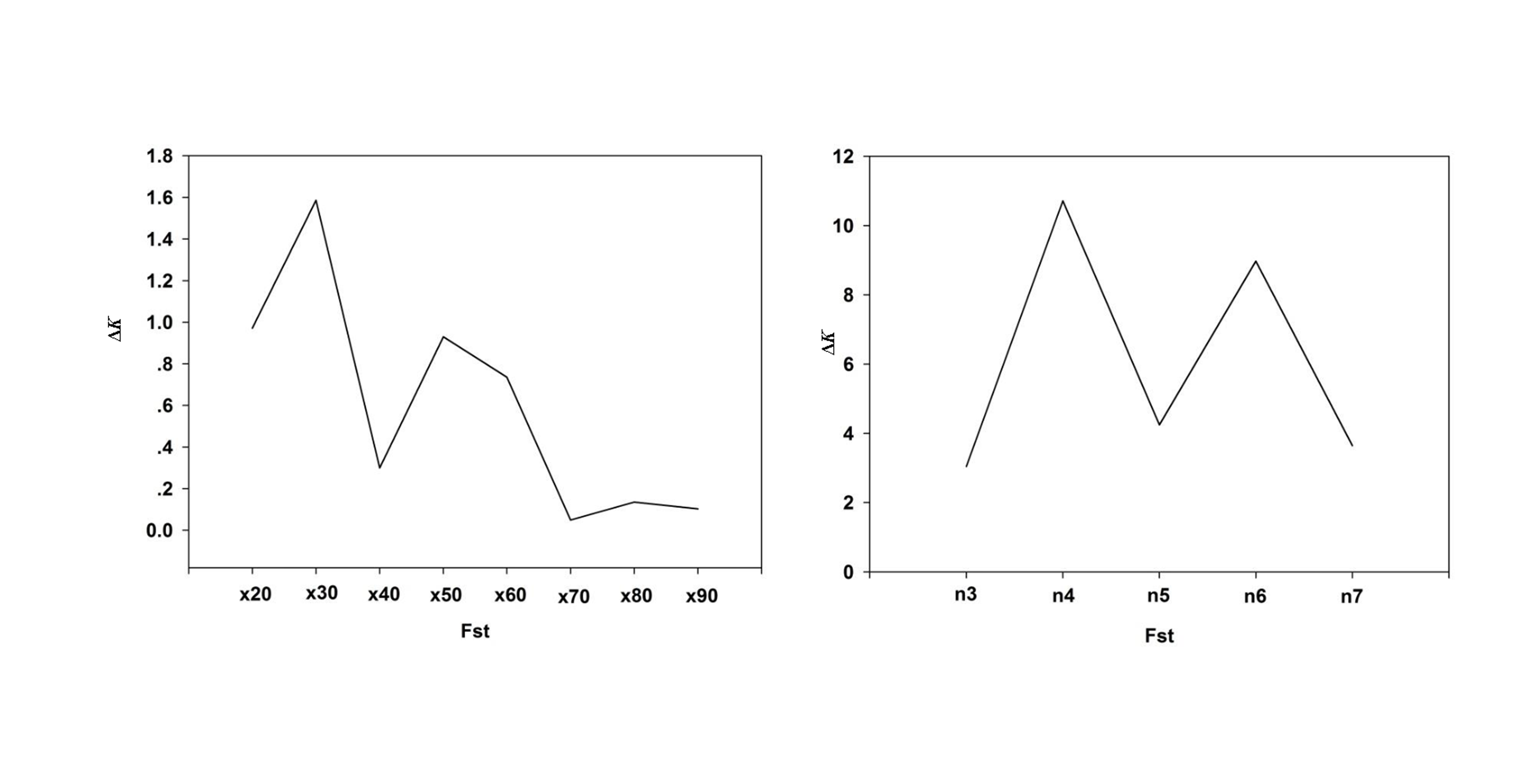

Supplement: Supplementary file 6 [file ECE3-10-38-s006.png]

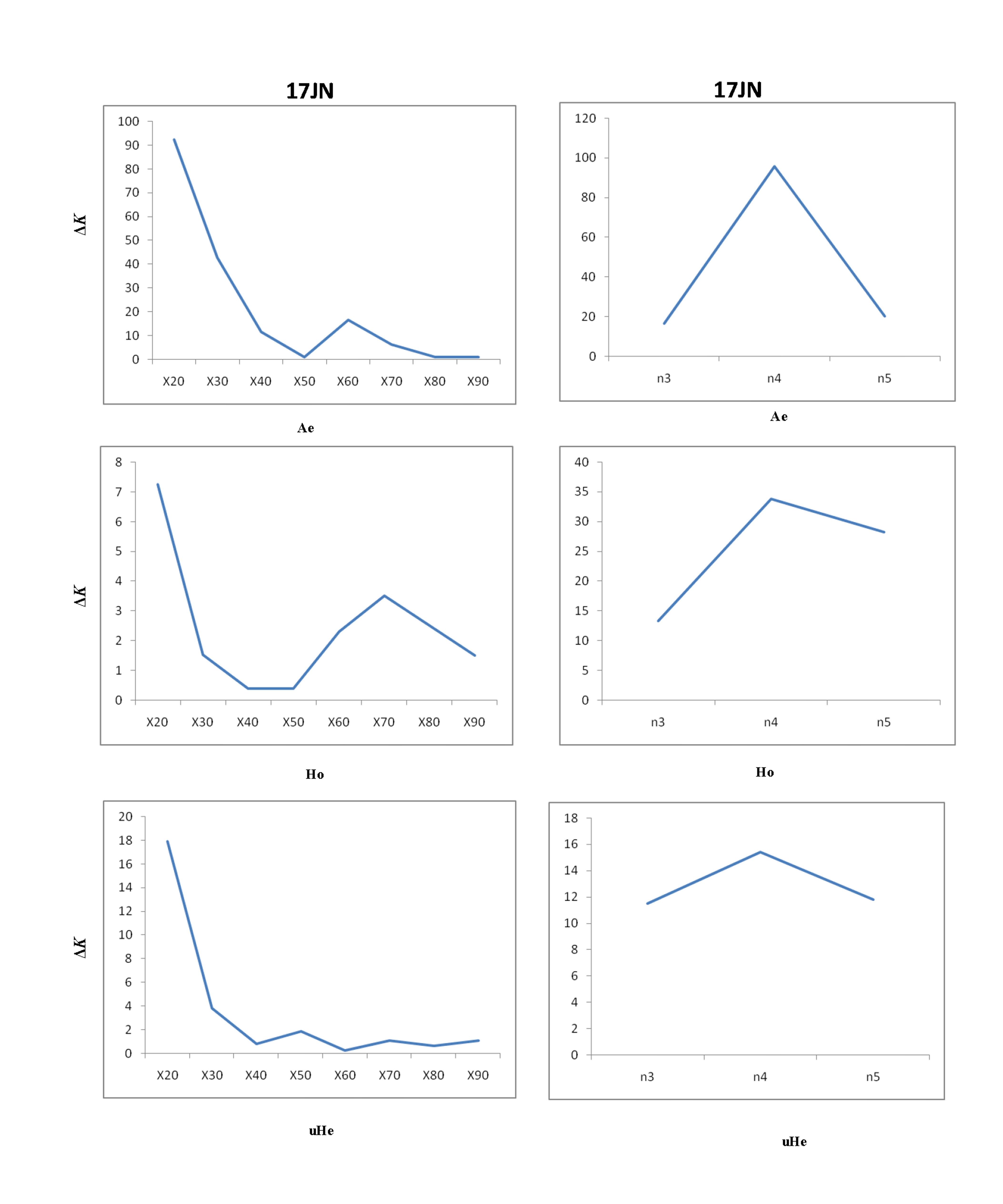

Supplement: Supplementary file 7 [file ECE3-10-38-s007.png]
