# Supplementary material for: Minimum sample sizes for invasion genomics: Empirical investigation in an invasive whitefly
Source: Ecol Evol. 2019 Oct 2;10(1):38–49. doi: 10.1002/ece3.5677 (PMC6972819; doi:10.1002/ece3.5677)
Supplement: Supplementary file 8 [file ECE3-10-38-s008.docx]

**FIGURE S1** Boxplots showing the minimum number of resampling replicates (x) needed to obtain accurate estimates of genetic diversity for populations of *Bemisia tabaci* (17JN and SPB), the boxplots on the left and right represent the results of 17JN and SPB separately. Center lines show the medians; box limits indicate the 25th and 75th percentiles; whiskers extend 1.5 times the interquartile range from the 25th and 75th percentiles; outliers are represented by dots; crosses represent sample means; bars indicate 95% confidence intervals of the means. *X* from 10 to 100 resampling replicates is the sample point. *Ae*, number of effective alleles; *H_O_*, observed heterozygosity; *uHe*, unbiased expected heterozygosity.

**FIGURE S2** Line charts showing the minimum number of resampling replicates (x) needed to obtain accurate estimates of genetic diversity for populations of *Bemisia tabaci* (17JN and SPB), and the line charts on the left and right represent the results of 17JN and SPB separately. The Δ*K* (y-axis) showed a peak at the optimal replicates (x). *Ae*, number of effective alleles; *H_O_*, observed heterozygosity; *uHe*, unbiased expected heterozygosity.

**FIGURE S3** Based on the optimal replicates (x) for populations of *Bemisia tabaci*, boxplots showing the minimum number of sample sizes (n) needed to obtain accurate estimates of genetic diversity for populations of *Bemisia tabaci* (17JN and SPB), the boxplots on the left and right represent the results of 17JN and SPB separately. Center lines show the medians; box limits indicate the 25th and 75th percentiles; whiskers extend 1.5 times the interquartile range from the 25th and 75th percentiles; outliers are represented by dots; crosses represent sample means; bars indicate 95% confidence intervals of the means. n is the sample size. *Ae*, number of effective alleles; *H_O_*, observed heterozygosity; *uHe*, unbiased expected heterozygosity.

**FIGURE S4** Line charts showing the minimum number of sample sizes (n) needed to obtain accurate estimates of genetic diversity for populations of *Bemisia tabaci* (17JN and SPB), the line charts on the left and right represent the results of 17JN and SPB separately. The Δ*K* (Y-axis) showed a peak at the minimum sample sizes (n). *Ae*, number of effective alleles; *H_O_*, observed heterozygosity; *uHe*, unbiased expected heterozygosity.

**FIGURE S5** Boxplots showing the optimal replicates (left) and minimum number of sample sizes (right) needed to obtain accurate estimates of *F_ST_* between populations of *Bemisia tabaci* (17JN and SPB). Center lines show the medians; box limits indicate the 25th and 75th percentiles; whiskers extend 1.5 times the interquartile range from the 25th and 75th percentiles; outliers are represented by dots; crosses represent sample means; bars indicate 95% confidence intervals of the means. x, resampling replicates; n, sample sizes.

**FIGURE S6** Line charts showing the optimal replicates (left) and minimum number of sample sizes (right) needed to obtain accurate estimates of *F_ST_* between populations of *Bemisia tabaci* (17JN and SPB). The Δ*K* (Y-axis) showed a peak at the optimal replicates and sample sizes. x, resampling replicates; n, sample sizes.

**FIGURE S7** Line charts showing the optimal replicates (left) and minimum number of sample sizes (right) needed to obtain accurate estimates of genetic diversity for populations of *Bemisia tabaci* (17JN). The Δ*K* (Y-axis) showed a peak at the optimal replicates and sample sizes. x, resampling replicates; n, sample sizes.
